# Bioethical knowledge in students and health professionals: a systematic review

**DOI:** 10.3389/fmed.2024.1252386

**Published:** 2024-04-10

**Authors:** Francisco Javier González-Blázquez, Antonio Ruiz-Hontangas, Clara López-Mora

**Affiliations:** Faculty of Health Sciences, Universidad Europea de Valencia, Valencia, Spain

**Keywords:** bioethics, training, assessment, healthcare professionals, ethical skill, bioethical competencies

## Abstract

**Introduction:**

Bioethics training is essential for healthcare professionals as it enables them to address ethical dilemmas in their clinical practice. However, there is still a lack of rigorous teaching programs, and assessing bioethical knowledge poses challenges.

**Methodology:**

Systematic review using the PRISMA method.

**Results:**

Analysis of 27 studies reveals a lack of ethical knowledge and skills among healthcare professionals and students. Specific training in bioethics is effective in developing bioethical competencies. Different approaches have been employed, including integrated training in academic curricula and intensive or ongoing programs. The results demonstrate improvements in knowledge, attitudes, and ethical values, although regularly updating these courses is recommended.

**Conclusion:**

Specific training, institutional support, and considering regional and disciplinary differences are necessary to enhance ethics in the practice of healthcare professionals.

**Systematic review registration:**

https://www.crd.york.ac.uk/prospero/display_record.php?ID=CRD42023437146, identifier CRD42023437146

## Introduction

The training of healthcare professionals is usually focused on the study and acquisition of knowledge aimed at developing diagnostic and treatment competencies. Likewise, it should also be linked to the development of humanistic skills that allow these professionals to practice their profession with a balance between the technical and the human aspects (1–4). This is highlighted by Striedinger (5) who argues that a framework of scientific and technical skills needs to be combined with a human dimension to address possible bioethical dilemmas that may arise in healthcare professions.

Training in bioethical aspects is a central and indispensable element that is progressively being included in the curricula of all health-related degrees. However, the training received is still inconsistent (5–7). This is mainly due to the need to approach the interactions between healthcare professionals and patients from a balanced technical and human perspective. While this may seem logical, evident, and indispensable nowadays, this reality is a recent consensus and has not always been a constant in health disciplines (8–10).

All of this is supported by Reich (11), who describes the process of evolution and development undergone by Bioethics, indicating that, from its early stages, it has drawn on moral, medical, and theological philosophy (11–13), enabling it to achieve the unified and scientific vision presented in Potter (14) work “Bioethics: The science of survival.”

Through literature, the consolidation of Bioethics as an independent discipline is evidenced by the use of scientific methods inspired by those used in the humanities and social sciences (14–18). With the development of bioethics, an empirical approach based on “principlism” has been adopted, such as the Belmont Report (autonomy, beneficence, non-maleficence, justice) (19), and other more inductive logics (20). While the consolidation of this discipline is already a fact, there is still progress to be made regarding the transfer of bioethical knowledge to healthcare professionals (21–24).

Given the tensions that exist between humanity and the practice of healthcare professionals regarding patients (25–28), coupled with uncertainties arising from modifications in healthcare systems (29, 30) and the impersonal advancement of new techniques, reflected in the substantial decrease, on the part of healthcare professionals, in the altruistic commitment to helping others (28–30), the importance of education in bioethics becomes evident in order to provide an optimal response in those moments when healthcare personnel may face ethical dilemmas (28, 31–33).

The humanization of healthcare services is directly related to the ethics, moral values, and professional deontology of healthcare agents toward the patient (34–36). Thus, bioethics seeks to combine humanism with the development of scientific knowledge, considering the patient not merely as a body or a medical process, but as a vulnerable human being facing illness (37–39).

The constant dissatisfactions of patients, who demand respect for their vulnerability in the face of illness, pose a challenge for healthcare institutions (34, 40–42). These institutions see bioethics as the link between health and humanization, reconciling clinical practice and the doctor-patient relational attitudes with ethical and moral reflection (43, 44). Therefore, knowledge and education in bioethics are essential to acquire more humane competencies and improve care while safeguarding the dignity and quality of life of patients, especially in situations of vulnerability (34, 45–47).

Similarly, bioethics is applicable in the administrative field of healthcare centers, aiming to provide patient care with greater quality and humanism (41, 48–50). This includes the establishment of an Assisting Ethics Committee in each healthcare sector, based in the reference hospital of the sector (Article 28 of BOE-A-2011-8403, Law 10/2011, of March 24, on the rights and guarantees of the dignity of the person in the process of dying and death).

In recent years, there has been a growing interest among the scientific and professional community in improving the education received by professionals in this aspect (51, 52). This is evidenced by the increasing, albeit limited, body of literature that points to the lack of rigorous teaching programs within university and professional contexts (6, 53–56), and that encourages the creation of specialized curricula in this area (56–62), in order to progressively develop the capacity to face ethical conflicts through simulated and real environments (6, 7, 53). On the other hand, experts recommend problem-based learning (PBL), which allows students to acquire not only theoretical content and knowledge (knowing and understanding), but also reflective and evaluative abilities (knowing how to act) and the necessary competencies to resolve different situations related to the profession (54, 63–66).

Espinoza Freire and Calva Nagua (67) and Carrera et al. (68) emphasize the need for this ethical education to start with the ethical training of academics since only through teacher education can the necessary knowledge be transmitted. This can be achieved through the implementation and design of new strategies that help mitigate the constant ethical dilemmas that arise in clinical practice and their consequences.

According to Culver (69), Bioethics education programs should not directly teach attitudes but rather focus on the identification of ethical conflicts that arise in clinical practice. Students should internalize the process for a rational response.

Couceiro-Vidal (54) highlights two main misconceptions regarding ethics education in healthcare. Firstly, the denial of freedom of conscience, and secondly, the presence of conflicts in the values due to the clinical relationship between healthcare professionals and patients and the paternalistic model that has been followed since ancient times.

Curriculum plans should take into account that moral development requires the development of schemas and different mental structures across six stages (obedience and punishment, individualism and exchange, interpersonal relationships, social order, social contract, and universal principles), which form three levels (pre-conventional, conventional, and post-conventional), as specified by León et al. (70), based on previous studies by Kohlberg (71). The latter two levels are where individuals seek the good for social and community wellbeing, understand that there are certain rules to be followed to live in a community, and use those norms to guide their actions in pursuit of the common good of their social group (71–73). At this level, individuals are capable of evolving toward full maturity of thought, establishing their own moral autonomy from which they can make absolute judgments of justice (70, 74–76).

In general, the academic and scientific community proposes a “common” model of aspects that would improve the method of teaching bioethics, subdividing it into “competencies to be achieved,” “knowledge,” and “skills” (54). It is suggested that in the preclinical period, basic bioethics should be taught, introducing students to the fundamental theoretical content. Then, in the clinical period, bioethics should be clinical, enabling students to learn skills to resolve specific conflicts that may arise in their clinical practice (66, 77–79).

### Assessment of bioethical knowledge

The assessment of competencies, attitudes, and behaviors that align with the values being conveyed in academic content remains challenging due to the novelty of the discipline and the multitude of application contexts (80, 81). In an effort to address this challenge, the academic and scientific community has sought to develop and implement existing examinations, such as the objective structured clinical examination (OSCE), as an evaluation methodology (82–84). The OSCE allows for the measurement of knowledge and the ability to ethically act in clinical situations (81, 85), but it is unable to assess learning in other areas, such as behaviors based on acquired ethical values (81, 86).

In this line, Couceiro-Vidal (54, 87), along with his proposal of problem-based learning (PBL), suggests a new evaluative method that allows for the objective assessment of bioethics learning in the clinical professional’s practice, acknowledging the complexity of the entire process and following a similar curriculum design structure as other subjects taught.

Vera Carrasco (88) explains that, for a proper evaluation of bioethics learning, it should be conducted in three specific periods. Firstly, a diagnostic assessment is conducted at the beginning of the course to determine the subject’s theoretical foundation. Secondly, during the course, there is a formative phase in which strengths and weaknesses in teaching should be identified. Lastly, a summative phase takes place at the end of the academic year, during which the instructor quantifies and grades the subject’s acquired knowledge. This third phase is crucial as it allows for the identification of weaknesses and enables the instructor and the evaluated individual to engage in self-assessment.

Of the available scales for this purpose, the Hirsch (89), Hirsch (90) for the evaluation of attitudes toward professional ethics is worth highlighting. It is based on research conducted by Escámez Sánchez (91, 92); Escámez Sánchez et al. (93), drawing from the ideas of Fishbein and Ajzens (94) “Theory of Reasoned Action” (1980), which conceives individuals as rational beings capable of judgment and evaluating situations (95, 96). This scale consists of 55 items that are responded to using a 5-point Likert scale (1–completely disagree; 5–completely agree) and allows for the assessment of cognitive competencies, social competencies, ethical competencies, and affective-emotional competencies. Another relevant scale is the “Problem Identification Test” developed by Hebert et al. (97), which aims to evaluate ethical knowledge in students, defining it as “the ability of a person to recognize the existence of a moral problem” (98–100). This instrument uses 4 clinical cases to semi-quantitatively assess the recognition of three fundamental principles of bioethics (Autonomy, Beneficence, and Justice).

However, most studies measuring theoretical and applied bioethical knowledge rely on ad-hoc scales with questions specific to each author (90, 101–104). In general, the design of questionnaires aimed at evaluating bioethical knowledge arises from professional experience and the needs faced by each teacher and/or area in bioethics (90, 105, 106). Hence, the importance of the teacher’s attitude in creating evaluative methods focused on resolving ethical dilemmas encountered in their professional practice.

Therefore, this research aims to demonstrate the level of knowledge in bioethical aspects among students and healthcare faculty, as well as promote critical reflection on bioethical education for improved practice.

## Materials and methods

### Study design

The methodological process was based on the recommendations presented by the PRISMA (Preferred Reporting Items for Systematic Review and Meta-Analysis) statement (107–110). All review phases were conducted in duplicate. The protocol for this study was registered in PROSPERO (International Prospective Register of Systematic Reviews) under the ID: CRD42023437146.

### Research strategy

The literature review was conducted between October and December 2022. To conduct the systematic review, a SPIDER framework (111) was employed. Within this framework, S (Sample) encompassed both students and healthcare professionals, PI (Phenomenon of Interest) focused on bioethical knowledge, D (Design) comprised descriptive or scale validation studies, E (Evaluation) centered on questionnaire outcomes, and R (Research type) encompassed quantitative studies.

The databases used were Web of Science, PubMed, PEDro, Lilacs, and Scopus. Additionally, specialized journals such as Bioethics in Health Sciences, Revista española de Bioética, Perspectivas Bioéticas, Revista latinoamericana de Bioética, Revista colombiana de Bioética, Revista Apuntes de Bioética, BioScientis, Bioética&Debate, Revista de Bioética y Derecho, Cuadernos de Bioética, Empirical Bioethics, Journal of Bioethics, Medicine and Bioethics, American Journal of Bioethics, and Journal of Medical Ethics were included. The following search terms were used in both English and Spanish: (bioethics OR deontology OR medical ethics OR ethics AND scale OR questionnaire OR validation OR evaluation AND health).

### Inclusion and exclusion criteria

To be included, studies had to meet the following criteria: (1) be published after 2019; (2) be written in English or Spanish; (3) provide previously unpublished original results; (4) aim to evaluate bioethical knowledge in the healthcare population or in training. Therefore, this work excluded literature reviews, systematic reviews, meta-analyses, books, general journals, editorials, comments on works, and articles that did not propose any intervention program and/or proposed it but not for the evaluation of bioethical knowledge in the healthcare population.

### Selection process

After completing the search in all sources, a total of two reviewers screened the abstracts of the obtained results, using the Rayyan support tool for the initial exclusion criteria. In cases where there were doubts, an independent professional expert in bioethics was consulted. The data collected from the accepted articles were grouped into a database for synthesis and further discussion in this document. The following data were extracted from the accepted articles: (1) primary authorship, (2) year of publication, (3) methodology used, categorized as qualitative or quantitative methods, (4) sample used, including age and origin of the sample if available, (5) type of evaluation or intervention, indicating the type of resource used, name, items, and questionnaire administration time if applicable, (6) overall assessment of bias risk, and (7) main study results.

Regarding the reduction of bias risk, this review proposes different strategies, including (1) addressing biases from the accepted studies in the review, (2) managing biases in the synthesis of the collected information, (3) addressing biases from articles that should have been included in the review but were not, and (4) addressing biases caused by conflicts of interest and/or authorship funding. To reduce the bias risk arising from the analysis, synthesis, and reflections generated by the authors of this document, maximum transparency has been provided in the selection method, coding, bias analysis, and information synthesis, allowing future replication by other professionals and promoting inter-rater validity. In order to reduce the bias risk associated with not admitting articles that could have been accepted for various reasons (e.g., not being published in an indexed journal, language barriers, gray literature), a comprehensive search strategy has been designed, including specialized journals.

## Results

**FIGURE 1:**
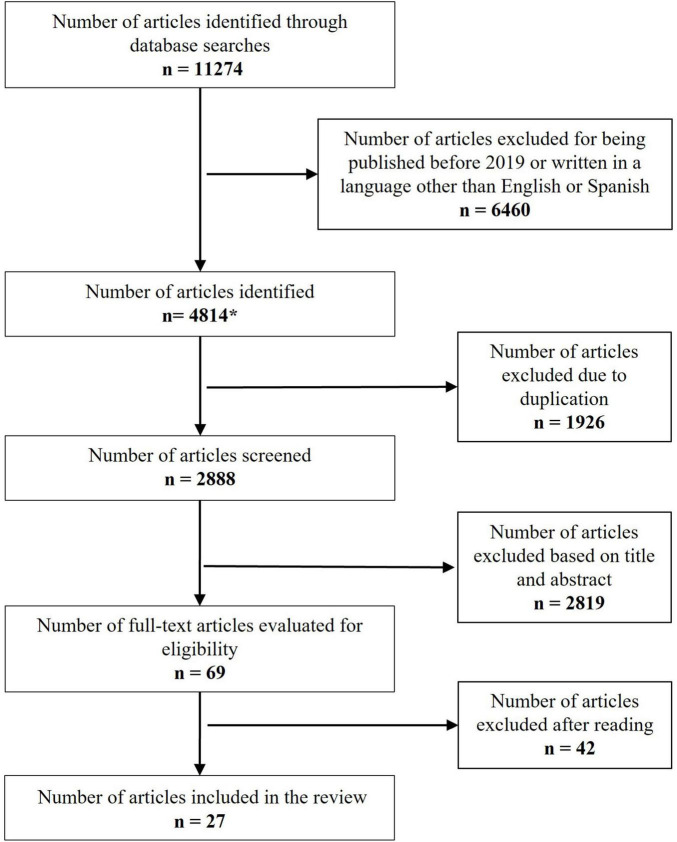
Flowchart. * = WOS (*n* = 1,825); PubMed (*n* = 906); Lilacs (*n* = 12); PedRo (*n* = 0); Scopus (*n* = 2,059); Rev. Colombiana de Bioética (*n* = 3); EIDON (*n* = 1); Rev. Latinoamericana de bioética (*n* = 2); Rev. Apuntes de bioética (*n* = 2); Rev. Bioética y derecho (*n* = 1); Theorical Medicine and bioethics (*n* = 2); Journal of Medical Ethics (*n* = 1).

Figure 1 shows the flowchart of the systematic review process. The literature search in databases yielded a total of 11,274 articles, out of which 6,460 were excluded for being published before 2019 or not being written in English or Spanish. Among the 4,814 identified articles, a total of 1,926 articles were discarded as duplicates. A total of 2,819 articles were rejected based on the title and abstract information; none of them were inaccessible, and the inter-rater consensus process was blinded. Therefore, a total of 69 articles were read in-depth and assessed for eligibility. Out of these, 42 articles were excluded from this study as their objective was not the evaluation of bioethical knowledge in the healthcare population. Finally, 27 articles were included in the present review.

### Main findings

#### Study characteristics

The main findings are summarized in Table 1. Out of the 27 reviewed articles, the majority were descriptive, specifically 15 had a cross-sectional descriptive design (5, 6, 7, 8, 11, 12, 13, 14, 15, 16, 17, 18, 20, 21, 26) and 2 had a longitudinal descriptive design (1, 23). On the other hand, 7 studies had a quasi-experimental design without a control group (2, 10, 24, 27) or with mixed methods (3, 4, 22). The remaining studies were experimental with a control group (9, 25) and validation of a scale (19).

**TABLE 1:** Summary of the reviewed studies.

Article number	References	Country	Objective	Sample	Design	Instruments	Results
1	Madigosky et al. (137)	USA	Demonstrate knowledge, skills, and behaviors of teamwork/collaboration, values/ethics, and quality/safety as a member of the interprofessional team. Demonstrate collaboration, teamwork skills, and behaviors as an interprofessional team. Identify the unique roles and responsibilities of each healthcare professional within the interprofessional team. Articulate a shared and interprofessional identity as a healthcare professional.	Students in initial-level programs of anesthesiology assistant, dentistry, medicine, nursing bachelor’s, pharmacy, physiotherapy, medical assisting, and public health programs. (2014)592- (2015)637- (2016)620	Longitudinal descriptive	Teaching method for bioethics in a transdisciplinary manner through group work. 16 sessions (8 at the end of the first semester + 8 at the end of the second). In the first 15 sessions, three aspects are addressed (collaboration, ethics, quality), and the 16th session involves applying everything previously discussed. An *ad hoc* questionnaire is administered at the beginning, middle, and end of the course. It consists of 34 Likert-scale questions and 3 open-ended questions.	Improvement of learning and skills both at the group and individual levels.
2	Perkins and Stoff (138)	USA	Describe the development and implementation of a specific pilot curriculum for pathology in bioethics.	*N* = 29 students in medical pathology (F? M?)	Longitudinal quasi-experimental study without a control group.	An *ad hoc* pre- and post-intervention survey was conducted. Five-hour sessions were held over the course of 14 months. Within the core curriculum, there was one introductory didactic session and three sessions focused on specific topics and case-based discussions. An additional second introductory session was conducted between sessions 2 and 3 to ensure that new medical pathology graduates from Emory were fully oriented to the curriculum objectives. The introductory sessions consisted of a 45-min to 1-h didactic presentation, which described the need for ethical education for pathology learners, basic terms and concepts, and the structure and objectives of the curriculum.	Most of the sample found the curriculum useful and learned something new after completing it. They also believed that a basic understanding and application of ethics and professionalism are essential to their current and future pathology practice.
3	De Panfilis et al. (117)	Italy	Develop a new specialized training program in medical ethics dedicated to a hospital’s UCP (Unit of Clinical Practice) along with its evaluation. Evaluate both quantitatively and qualitatively the impact of the training on students in terms of enhancing their ethical skills	*N* = 8 (F? M?) The participants of the training program are employees of the Public Hospital for Oncological Research in Reggio Emilia. The group consists of 3 doctors, 2 nurses, and 3 psychologists.	A mixed-methods design with pre- and post-intervention evaluation	The training program has been named “Teach for Ethics in Palliative Care” (T4EPC). It involves 28 h of training conducted over 36 weeks. The training focuses on a theoretical session (8 h in three meetings), a theoretical-empirical session (10 h in three meetings), and a session centered on individual ethical consultation upon request (10 h in 5 meetings). The assessment of learning follows the Moore model, ranging from Attendance (Level 1) to Change in Practical Performance (Level 5). Data were collected through semi-structured interviews (participant expectations), an ethical skills portfolio, and an analysis of an ethical case.	The results highlight those participants developed deeper ethical knowledge and awareness. They also felt more confident and motivated to widely apply ethical reflections and reasoning in their daily practice.
4	Naseem et al. (139)	Pakistan	To test the effectiveness and feasibility of a mobile learning app (EthAKUL) for teaching bioethics in a university in Pakistan using the M-JiTL (Mobile-Just-in-Time Learning) method.	*N* = 67 (pre-intervention) (F = 48; M = 19) *N* = 29 (post-intervention) (F = 17; M = 12) The average age is 25 years.	A mixed-methods design with pre- and post-intervention evaluation	A knowledge test was designed and administered before and after the intervention to assess changes in students’ bioethics knowledge. The pre-test was completed by students during orientation workshops, while the post-test link was emailed to them one week after the intervention had concluded.	Changes in bioethics knowledge were measured by comparing the pre- and post-test results. A significant change (*p* = 0.012) was observed in the overall mean score of the pre-intervention bioethics knowledge test for the students (9.34 ± 2.37), and the post-intervention mean score (10.38 ± 1.98), indicating an increase in the students’ knowledge scores. No significant changes in mean scores were found based on gender.
5	Tekleab and Lantos (140)	Ethiopia	Explore the ethical knowledge, attitudes, and experiences of physicians in a pediatric referral hospital in Addis Ababa, Ethiopia, a resource-limited setting.	*N* = 59 participants completed the questionnaire (F = 36; M = 23) Mean age 30.7 years.	Descriptive correlational study.	An *ad hoc* questionnaire was designed to address the following characteristics of the respondents: demographic characteristics, knowledge about certain domains of pediatric bioethics (maximum score of 23, +19 indicating good knowledge), attitudes, and experiences of ethical dilemmas encountered by clinicians during their practice (9 Likert-scale questions, maximum score of 45).	The findings indicated that the respondents had poor knowledge of many important ethical principles. The average knowledge score of the respondents (12.3) was lower than that reported for pediatricians in the United States, where the average knowledge score was 17.3. This suggests that modern bioethical principles may conflict with traditional practices in certain countries. It also suggests that education is likely to be effective in changing knowledge, beliefs, and attitudes
6	Jahan and Flora (141)	Bangladesh	Evaluate the attitude of newly graduated medical students toward medical ethics and professionalism.	*N* = 308 (F = 144; M = 164) Mean age = 24.2 years	Descriptive correlational study.	An *ad hoc* questionnaire using a 5-point Likert scale was administered.	51.6% of the respondents emphasized the importance of ethical conduct and patient autonomy, but there still exists a significant paternalistic attitude (88.6%). The mean scores for the maximum statements were around 3, indicating that individuals may not express themselves as confidently as expected. The majority of the respondents (85.4%) favored the inclusion of a mandatory module on medical ethics and professionalism for the improvement of their practice and knowledge.
7	Arslan et al. (142)	Turkey	Analyze the relationships between moral development and ethical decision-making in nurses.	*N* = 227 nurses (F = 187; M = 40). Ages ranging from 20 to 50 years.	Descriptive correlational study	The Nursing Dilemma Test, sociodemographic form, and the Scale of Moral Development for Professionals were used. The Scale of Moral Development consists of three factors: pre-conventional level, conventional level, and post-conventional level. It includes 12 items and is measured using a Likert-type scale. The scale’s minimum score is 12, and the maximum score is 60. Higher scores indicate a higher level of moral development. The scale scores are categorized as follows: 12–27 indicates pre-conventional level, 28–44 indicates conventional level, and 45–60 indicates post-conventional level.	In this study, it was found that nurses were at the post-conventional level according to Kohlberg’s theory of moral development. Sociodemographic and work-related characteristics did not affect their scores in moral development level and scores in nursing-based principle thinking, practical consideration, and familiarity (*p* > 0.05). Nurses pay attention to moral principles during decision-making, although not at a desirable level, and they are relatively influenced by environmental factors.
8	Maluwa et al. (114)	Malawi	Analyze the level of ethical competence among clinical nurses working in selected hospitals in Malawi; Identify the determinants of high-level ethical competence; Describe the indicators/characteristics of ethical competence.	*N* = 271 (235 responded) (F = 180, M = 55) Age range between 21 and 40 years.	Descriptive cross-sectional	The questionnaire consisted of three parts: Demographic data Level of competence in ethics (The Moral Competence Scale for Home Care Nurses–MCSHCN). This scale consists of 45 items based on five theoretical components of moral competence, which are moral/ethical sensitivity, moral/ethical judgment, moral/ethical motivation, moral/ethical character, and implementation of moral/ethical decisions. Characteristics of ethical competences. This question allows for open-ended responses to express themselves accurately in their own words.	The results showed that there was no significant difference (*p* > 0.05) between demographic characteristics and the level of ethical competence. This study has confirmed that the MCSHCN is a reliable instrument for measuring ethical competence among nurses and midwives in resource-limited countries like Malawi. The scores of clinical nurses in this study ranged from 3.16 to 5, indicating that all clinical nurses were ethically competent.
9	Nesime and Belgin (143)	Turkey	Evaluate the effectiveness of the nursing education curriculum in providing knowledge, attitudes, behaviors, and ethical sensitivity in advocating for, developing, protecting, and maintaining health.	*N* = 80 nursing students (2 groups of 40) F = 36, M = 4 Mean age = 21.3 years	The study is a pre-test, post-test, parallel group, randomized controlled study (RCT).	Social Justice Advocacy Scale (SJAS) The Moral Sensitivity Questionnaire (MSQ)	On the Ethical Sensitivity Scale, the pretest scores of the experimental group and the control group were similar (*p* > 0.05). The posttest score of the experimental group was significantly higher than their pretest score and the posttest score of the control group (*p* < 0.001).
10	Hertrampf et al. (144)	Germany	Evaluate the attitudes toward ethical issues affecting dental students at the School of Dentistry in Kiel during patient treatment.	*N* = 23 (F = 18 M = 5)	Qualitative study	Standardized semi-structured interviews were conducted.	None of the students exhibited relevant theoretical knowledge in the field of medical ethics or skills for ethical conflict resolution.
11	Macpherson et al. (145)	Spain	Evaluate ethical decision-making during the early stages of student training.	*N* = 294 students (F = 184, M = 112)	A mixed-methods study using narrative responses to a case with ethical implications in the field of gender-based violence.	A procedure was developed through Case-Based Learning (CBL) in 30 h of seminars. An *ad hoc* questionnaire was used to assess knowledge.	The results indicate significant differences in responses between specialties based on scores on ethical knowledge tests. No significant differences were found between the responses provided by men and women. Instead, four categories of responses were identified because of combining personal conversation, reporting to legal authority, or seeking assistance from other teams. The most common option among dentists is only conversation, while physiotherapists include assistance from other teams. In nursing, a balance between both possibilities is observed.
12	Feller et al. (115)	USA	Identify if there are differences in nursing professional values based on program type and/or geographic location.	*N* = 417 nursing students	A descriptive cross-sectional study.	The Nursing Professional Value Scale-Revised (NPVS-R) is a scale consisting of 26 descriptive statements reflecting a particular disposition of the code of ethics, including its interpretation found in the ANA Code of Ethics (2001). It uses a five-point Likert scale generating scores ranging from 26 to 130; higher scores represent a greater assimilation of strong professional values.	The results indicate that pre-licensure nursing students are educated with values integrated into the nurses’ code of ethics. Significant differences (*p* < 0.05) were found when comparing geographic locations, program types, and scores on the factors of the Revised Nursing Professional Value Scale.
13	Paşalak et al. (112)	Turkey, Tanzania, & Spain	Analyze the professional values of nursing students from different countries.	*N* = 305 (F = 221, M = 84) Mean age = 23.4 years	Comparative descriptive study.	Nurses’ Professional Values Scale–Revised.	The levels of ethical values and professionalism among Turkish and Spanish students were similar but higher than those of Tanzanian students. Among Turkish students, female students who were single and whose parents had a high level of education obtained higher scores in professional values compared to others.
14	Bleda et al. (116)	Spain	Analyze nursing students’ perceptions of professional values throughout the 4 years of education.	*N* = 315 nursing students	Cross-sectional descriptive study.	The EVPS (Nursing Professional Value Scale Revised) is a self-administered instrument consisting of 26 items, divided into three dimensions: ethics, professional competence, and professional mastery. Each response is provided using a 5-point Likert scale of importance: (1) not important at all, (2) somewhat important, (3) important, (4) very important, and (5) extremely important. The respondents selected the degree of importance they assigned to each nursing practice value statement.	The students’ perceptions of professional values were found to be significantly correlated with their academic year. Overall, the students scored higher in the ethics dimension. This suggests that as students progress through their nursing education, they develop a stronger understanding and importance placed on ethical considerations in their professional practice.
15	Barman et al. (146)	India	Analyze the ability to recognize different bioethical issues in relation to patient care Analyze the ability to recognize changes in the pattern of bioethical issue recognition after formal training	*N* = 50 medical and nursing students (MBBS) (F = 22, M = 28) Ages between 20 and 22 years	Cross-sectional study	Self-administered questionnaire. Each question on a Likert scale, with a minimum score of 1 (1 = strongly disagree) and a maximum score of 5 (5 = strongly agree). After 6 months of training and clinical exposure, the students were re-evaluated using the same questionnaire.	All respondents in the study group agreed that medical ethics is highly important, but only 24% were aware of the existence of an ethics committee at the institute. Changes were observed after clinical exposure in responses such as disclosing the patient’s condition to close relatives (54 to 84% agreement before and after exposure, respectively) and discussing ethical issues related to clinical cases (74 to 94% agreement before and after exposure, respectively). Some issues remained unclear even after clinical exposure, such as doctors refusing to perform an abortion (56% disagree and 38% agree), consent for treatment in children (60% disagree and 32% agree), and the use of brand-name medications versus generics (76% generics and 26% brand-name).
16	Ashfaq et al. (147)	Pakistan	Evaluate the basic knowledge and perception of medical students regarding bioethical issues in clinical practice following their exposure to formal bioethics education in their curriculum	*N* = 285 students (F = 196, M = 89) Mean age 21–23 years	Cross-sectional study	Self-administered questionnaire that included multiple-choice and scenario-based questions related to ethical dilemmas encountered during clinical practice. 5-point Likert scale.	Overall, 63% of students had adequate knowledge of bioethics. Medical students from private universities (57%) had slightly better knowledge of bioethics than their counterparts from public universities (43%).
17	Althobaiti et al. (148)	KSA	Evaluate the knowledge, attitude, and medical ethics in nurses	*N* = 1,943 nurses and technicians F = 63.1% M = 36.9%	Descriptive cross-sectional study	Ad hoc questionnaire developed based on previously published literature to collect demographic data, position, duration of practice, prior study of medical ethics, previous training in bioethics, presence of an ethics committee in the institution, and previous experience of an ethical issue and how it was addressed. The questionnaire included items on participants’ knowledge, attitude, and practice related to care ethics.	Specialist/nursing technicians with 20- < 30 years of experience and female participants with prior training in bioethics had significantly higher average attitude scores than others.
18	Sinha et al. (119)	India	Evaluate the knowledge of ethics among young students and professionals, and the ethical practices in healthcare among medical professionals in a government university hospital in India.	*N* = 84 doctors, postgraduates, and consultants. Average age: 20–24 years	Cross-sectional study using convenience sampling	Data were collected through a structured and validated self-administered questionnaire consisting of 27 items on knowledge, beliefs, and attitudes toward the principles and practice of bioethics in clinical research. The questionnaire was administered before and after a conference/seminar on ethical principles.	Based on the pre- and post-workshop assessment, there is a significant need to emphasize ethical principles and review these concepts. Workshops and interactive sessions are a good means for periodic evaluation and reinforcement of these values in our research and clinical practice. Therefore, they should be included in the curriculum of all educational institutions.
19	Chughtai et al. (149)	Pakistan	Develop an instrument to assess the ethical sensitivity of newly licensed dentists.	*N* = 107 (F = 70, M = 37) Mean age = 23.7 years	Instrument development study (IDS)	Dental Ethical Sensitivity Scale (DESS).	No significant relationship between gender and ethical sensitivity. The scale can be used locally to assess newly licensed dentists and enhance their cognitive ethical decision-making.
20	Esquerda et al. (150)	Spain	Assess the impact of ethics education by measuring the evolution of Kohlberg’s moral reasoning and ethical sensitivity in resolving clinical cases.	*N* = 175 third-year medical students (78 before taking bioethics and 97 after taking bioethics, in different courses). (F = 126; M = 45; missing = 4) Mean age = 20.8 years	Cross-sectional observational study.	A sociodemographic questionnaire, Rest’s Defining Issue Test as a measure of moral reasoning, and Hébert’s Problem Identification Test as a measure of ethical sensitivity were administered.	No differences are found in the moral development of medical students before and after formal education in bioethics, but differences are observed in case resolution skills. Females exhibit higher post-conventional reasoning, indicating greater moral development.
21	Palanisamy and Xiong (151)	USA	Reinforce and enhance the practical knowledge of medical ethics students regarding patient capacity assessment and discharge planning in the context of acute neurological impairment.	*N* = 23 3rd-year medical students.	Cross-sectional study.	First, they completed a brief pre-test consisting of five questions to measure their prior knowledge of the learning objectives of the activities. Then, the students participated in a 1-h interactive session, facilitated by an instructor, in small groups. After the activity, we assessed their knowledge again using a five-question questionnaire. Pre-post test questionnaire	Qualitatively, students reacted positively to the interactive activity, and the pre- and post-test scores demonstrated an improvement in their factual knowledge of the activity’s objectives (+40%). The innovative use of an interactive teaching method proved effective in achieving our educational goals.
22	Kenny et al. (152)	Australia	Analyze the effectiveness of Ethics in Professional Practice (EPP) (simulation) training for healthcare ethics in students.	*N* = 81 voluntary students from Health professions in Australia. *n* = 12 Exercise Physiology *n* = 16 Rehabilitation Counseling *n* = 53 Speech Pathology	Quantitative and qualitative study.	Learning measured by Semantic Differential Scales (pre-post EPP). Behavior measured by clinical case vignettes. Effects measured using a rubric. Kirkpatrick Model (reaction, learning, behavior, and effects).	Improvement observed in concepts such as collaboration, communication, and quality of care.
23	Wall (120)	USA	Analyze the effectiveness of an ethics training seminar (75 min) in oncology nurses.	*N* = 107 oncology nurses	Longitudinal descriptive panel study	*Ad hoc* knowledge test with 18 multiple-choice questions	A significant short- and medium-term improvement in knowledge acquisition was observed. In the long term, knowledge tends to decline. Auditors recommend continuous training.
24	Mosalanejad et al. (153)	Iran	Design a blended learning program based on a constructivist approach to ethical reasoning and determine its effect on students’ reflection and learning.	*N* = 35 medical ethics students	Quasi-experimental study with a single-group pretest-posttest design	Self-Reflection and Insight Scale (SRIS) Objective structured clinical examination (OSCE) test (with TOSCE)	The blended constructivist approach may have a favorable effect on students’ clinical reasoning. Thus, the model appears to be an appropriate method for teaching medical ethics and resolving ethical conflicts.
25	Momennasab et al. (121)	Iran	Analyze the impact of group training on nurses’ knowledge, attitude, and performance regarding ethical codes.	86 nurses. Intervention Group: *N* = 44, Mage = 30.15 (4.96); Control Group: *N* = 42, Mage = 30.95 (5.17)	Quasi-experimental study with a control group.	Knowledge tests based on *ad hoc* ethical codes and Iranian nursing. Ethics Codes; *ad hoc* attitude rating scale; *ad hoc* performance questionnaire.	After the training, nurses showed improvements in attitude and skills scores but not in knowledge.
26	Pais et al. (154)	India	Assess the knowledge, practice, and attitudes of postgraduate students in medicine, dentistry, and physiotherapy toward healthcare ethics.	*N* = 60 postgraduate students in medicine, dentistry, and physiotherapy. Mean Age: 26.23 ± 2.33	Descriptive correlational study.	*Ad hoc* created questionnaire on healthcare ethics.	Postgraduate students in dentistry scored lower in their knowledge levels compared to postgraduate students in physiotherapy and medicine.
27	Geis et al. (118)	USA	Develop and test a digital curriculum on ethics and professionalism in neonatology, and analyze the effects on students’ knowledge and confidence.	*N* = 49 neonatology students Mean age = 32.3 years (SD 3.0)	Quasi-experimental longitudinal panel study	Kirkpatrick model; TEKNeo; Trust and Competence Test	A significant improvement was observed in general ethical knowledge and confidence. Furthermore, there were significant improvements in the principles of bioethics, maternal-fetal decision making, prenatal counseling, genetic screening, justice and social issues, resource allocation, rights and ethics, morality, and medical errors.

Regarding the countries/geographical areas represented in the review, the samples were mainly composed of residents from the United States (1, 2, 12, 21, 23, 27) and Europe (11, 13, 14, 20, 3, 10), followed by samples from Pakistan (4, 16, 19), Turkey (7, 9, 13), India (15, 18, 26), Iran (24, 25), Ethiopia (5), Bangladesh (6), Malawi (8), Tanzania (13), Saudi Arabia (17), and Australia (22). Except for the study led by Paşalak et al. (112), which explores a cross-cultural analysis of professional ethical values among nursing counterparts from Turkey, Spain, and Tanzania, the rest of the studies had samples from a single country.

The fields of study in the healthcare domain were diverse. 89% of the studies included nursing professionals (3, 4, 7, 8, 9, 11, 12, 13, 14, 15, 17, 23, 25) and medical professionals (1, 2, 3, 4, 5, 6, 15, 16, 18, 20, 21, 24, 26, 27), while 11% involved professionals from other healthcare disciplines such as dentistry (1, 10, 11, 19, 26), physiotherapy (1, 11, 22, 26), pharmacy (1), psychology (3), speech therapy, and sports sciences (22). Additionally, 26% of the studies included multiple healthcare disciplines (1, 3, 4, 11, 15, 22, 26).

To quantify knowledge in bioethics and related outcomes, ad-hoc questionnaires were used in 60% of cases (1, 2, 3, 5, 6, 10, 11, 15, 16, 17, 18, 21, 23, 25, 26, 27). Among studies that employed standardized (113) scales, the Nursing Professional Values Scale-Revised (NPVS-R) was used in three studies, specifically designed to measure altruism, autonomy, knowledge, ethics, integrity, and justice in nursing professionals (12, 13, 14). The Kirkpatrick protocol was used in conjunction with the Semantic Differential Scale (22) or the TEKNeo (27) in two studies. One study combined the Objective Structural Clinical Examination (OSCE) with Self Reflection and Insight (SRIS) (24). Another study used the Social Justice Advocacy Scale and Moral Sensitivity Questionnaire together (9). The Nursing Dilemma Test and Moral Development Scale for Professionals were used together in another study (7). Similarly, the Defining Issue Test and Problem Identification Test by Hebert were used in conjunction in one study (20). Additionally, the Dental Ethical Sensitivity Scale (19), The Moral Competence Scale for Home Care Nurses (MCSHCN) (8), and semi-structured interviews (3, 10) were found.

The objectives outlined in the studies were diverse. Nearly half of the reviewed articles (2, 4, 9, 15, 20, 21, 22, 23, 24, 25, 27) aimed to evaluate the effectiveness of bioethics education through curriculum implementation or specific training. On the other hand, six studies focused on assessing knowledge in ethics, as well as individuals’ attitudes and competencies related to it (1, 5, 8, 17, 18, and 26). The study by Maluwa et al. (114) went further by attempting to identify determinants of adequate ethical competence. Four studies sought to analyze the reflection process associated with decision-making in ethical dilemmas present in healthcare practice (6, 7, 10, 11). Furthermore, three studies aimed to explore differences in professional values among different training programs or geographical locations (112, 115, 116).

#### Bioethical knowledge

The analysis of the studies demonstrates that, in general, there is insufficient knowledge in the field of medical ethics and/or skills for resolving ethical conflicts among healthcare professionals and students. There is also a perceived lack of support from universities and workplaces, and there is consensus on the need to incorporate mandatory training in professional ethics (6, 7, 8, 10). However, it is observed that work experience and level of education completed are associated with an improvement in knowledge and ethical values (13, 14, 17, 20).

Despite this, in terms of ethical competence or knowledge, there do not seem to be significant differences based on gender or professional role (4, 7, 8, 11, 13, 19). However, it is important to note that, regarding the selected outcome variable, these results are not consistent across all studies. Some studies show that women have higher postconventional moral reasoning (17, 20), which is associated with a higher level of ethical competence in professional practice. There are also cases where dental professionals have lower ethical knowledge compared to their counterparts in nursing, medicine, or physiotherapy (11, 26).

On the other hand, significant differences exist in terms of bioethical knowledge, attitudes, values, or professionalism based on the geographical region of reference. Developed regions, such as the United States, tend to show higher scores than less developed regions like Malawi or Ethiopia (6, 12, 13). The same applies when comparing education in public and private universities, where students from private universities tend to achieve higher scores in ethical knowledge (16).

#### Characteristics and effectiveness of training

Out of the 27 articles analyzed, 16 included bioethics training as an independent variable, and the majority of them demonstrated that specific bioethics training is effective in developing bioethical knowledge, attitudes, values, or competencies for professional practice. These bioethics trainings showed significant heterogeneity in terms of their format and duration.

In the case of undergraduate student training, 4 studies proposed integrated training programs within academic curricula (2, 9, 20, 24), while 6 studies presented complementary programs external to the curriculum (1, 4, 9, 11, 15, 21, 22). In the case of integrated training within the curriculum, the training combined theory and practice, utilizing clinical cases, vignettes, discussion forums, simulation scenarios, among others. For trainings outside the academic curriculum, the proposed programs had a shorter duration (between 3 and 16 sessions) and were characterized by experiential learning through didactic tools such as problem-based learning (PBL and CBL), case dramatization, lectures delivered by renowned professionals, problem-solving in complex simulation scenarios, or the use of mobile applications.

Training programs for practicing professionals exhibit a variety of structures, but all of them are equally effective. It is possible to distinguish between intensive proposals and ongoing proposals over time.

Among the ongoing proposals, an example is the training program called “Teach for Ethics in Palliative Care” (T4EPC) proposed by De Panfilis et al. (117). This program consists of 28 h of training distributed over several weeks. During this time, professionals receive theoretical training (8 h), practical training (10 h), and individual mentoring (10 h). This training approach has proven to be effective in improving the professional practice of physicians, nurses, and psychologists who have completed the program.

On the other hand, the program developed by Geis et al. (118) consists of 13 ethics modules targeted at neonatology fellows. This program has been effectively tested in three academic institutions using a flipped classroom approach. The modules cover a wide range of topics in bioethics, such as principles of bioethics, maternal-fetal decision-making, professionalism and communication, prenatal counseling, withholding or withdrawing life-sustaining treatment, cultural sensitivity, genetic screening, palliative care, social justice and resource allocation, law and ethics, moral dilemma and physician awareness, disclosure of medical errors, and research ethics.

Intensive programs have also proven to be effective in developing ethical competencies. Sinha et al. (119) proposes a theoretical seminar that addresses knowledge, beliefs, and attitudes related to principles and clinical practice. This approach successfully improves the knowledge of participating medical professionals who underwent the program.

On the other hand, Wall (120) suggests a 74-min didactic seminar targeted at oncology nurses. Techniques such as storytelling, role-playing, and simulation are used in this seminar. The presented stories illustrate the role of oncology nurses in protecting and advocating for vulnerable patients, respecting and adapting to cultural differences, and increasing self-awareness of personal values that may influence decisions. According to the findings, there is a significant short- and medium-term improvement in the ethical competencies of nurses. However, it is suggested that these trainings need to be regularly renewed and updated as a stagnation in long-term improvement has been observed.

Furthermore, Momennasab et al. (121) propose bioethics training for nurses through independent reading of cases that present various ethical conflicts in relation to the professional code of ethics, followed by group reflection. This approach shows improvement in attitude and ability to resolve ethical conflicts, although no significant improvement is observed in ethical knowledge.

## Discussion

This review summarizes the findings of studies that address the analysis of bioethical knowledge in healthcare students and professionals, as well as the perception of knowledge and ethical competencies in healthcare professionals and students. Twenty-seven studies were systematically reviewed, all of which demonstrate the reality of existing bioethical knowledge and education in healthcare settings. To distinguish between the reviewed studies and other evidence, the reviewed studies will be cited using the assigned numbers in Table 1.

### Findings on bioethical knowledge and education in the field of healthcare

Overall, the evidence from the reviewed studies suggests that education in ethics and bioethics in the healthcare field is a topic of increasing interest, both in academic and professional contexts. This is because healthcare professionals constantly face complex ethical situations in their daily practice.

Drawing conclusions regarding the competence of healthcare students and professionals in terms of knowledge and skills to address bioethical dilemmas is challenging, as the analysis of the studies presents conflicting results. On one hand, there are studies that reveal a lack of sufficient knowledge in the field of medical ethics and skills for ethical conflict resolution among healthcare professionals and students (6, 7, 10), while others conclude adequate levels of knowledge in this population (8, 16). These results are consistent with what has been pointed out by Bellver Capella (122), Suárez Alba and Artiles Chaviano (123), and they confirm the ongoing difficulty in drawing solid conclusions due to the disparity of theoretical conceptualizations and procedures employed in different studies, as well as the limited representation of healthcare professions other than medicine or nursing.

Despite the existing deficiencies, it is observed that work experience and the level of completed education appear to be associated with an improvement in knowledge and ethical values (13, 14, 17). This indicates that time and exposure to ethical situations in professional practice can contribute to the development of ethical competencies (124–126). Additionally, there is evidence of a perceived lack of support from universities and workplaces in the development of ethical competencies. This deficiency is reflected in the consensus on the need to incorporate mandatory training in professional ethics (28, 31–33).

When analyzing differences based on gender and professional role, heterogeneous results have been found. In some studies, no significant differences have been identified in terms of ethical competence or knowledge (4, 7, 8, 11, 13), following the findings of authors such as Coffin-Cabrera et al. (127) or Sanz Ponce and Hirsch Adler (128). However, in other cases, it has been observed that women exhibit higher postconventional moral reasoning, which has been associated with a higher level of ethical competence in professional practice (17, 20), as stated by Barba (129) and Barba and Romo (130). On the other hand, it has been observed that dental professionals show a lower level of ethical knowledge compared to their nursing, medical, or physiotherapy counterparts (11, 26). These differences may be influenced by specific contextual and educational factors of each profession, as similarly asserted by García-Vilanova and Pérez (6), Nicoletti et al. (7), and Striedinger (5).

Another relevant aspect identified in the conducted review is the influence of geographical region and university type on ethical knowledge and competencies. Studies indicate that more developed regions, such as the United States, demonstrate higher scores in professional ethics compared to less developed regions like Tanzania or Ethiopia (6, 13). Additionally, it has been found that students studying at private universities achieve higher scores in ethical knowledge compared to those studying at public universities (16). These differences may be related to the availability of resources and the educational approach adopted in each context (130–133).

### Methodological limitations in research on bioethical knowledge in the health field

In the context of ethics and bioethics training strategies, there is variability in terms of approaches and durations. The examined studies used both ad-hoc training and standardized scales to assess knowledge and ethical outcomes. In most cases, ad-hoc questionnaires were implemented to measure ethical knowledge (1, 2, 3, 5, 6, 10, 11, 15, 16, 17, 18, 21, 23, 25, 26, 27). Additionally, specific scales (113) such as the Nursing Professional Values Scale-Revised (NPVS-R) were employed to evaluate ethical values in nursing professionals (12, 13, 14), which consists of six main dimensions: altruism, autonomy, knowledge, ethics, integrity, and justice.

The objectives set in the studies were also diverse. One of the most common objectives was to evaluate the effectiveness of bioethics training through the implementation of curricula or specific training programs (2, 4, 9, 15, 20, 21, 22, 23, 24, 25, 27). Additionally, the aim was to assess the knowledge, attitudes, and ethical competencies of individuals (1, 5, 8, 17, 18, 26) and analyze the reflective process associated with ethical decision-making in healthcare practice (6, 7, 10, 11).

Overall, the results indicate that specific bioethics training is effective in developing ethical knowledge, attitudes, values, and competencies in both students and practicing professionals. Both integrated curriculum-based training and external supplementary training have proven to be effective. These trainings combine theory with practice, utilizing didactic tools such as clinical cases, vignettes, discussion forums, simulation scenarios, and mobile applications.

Furthermore, training targeted at practicing professionals has also demonstrated their efficacy. Intensive and long-term programs have been proposed. Some intensive programs focus on theoretical seminars, while others adopt more participatory approaches such as storytelling techniques, role-playing, and simulation. These programs have successfully improved the ethical competencies of physicians and nurses, as well as promoted reflection on personal values that may influence ethical decisions.

However, it is important to highlight the need for ongoing updating and renewal of these training. It has been observed that, in the long term, stagnation in results may occur, indicating that ethical training should be a continuous and dynamic process to ensure its effectiveness over time (23). This idea aligns with the views of authors such as Alarcón and Chapa (134), Tarzian and Asbh Core Competencies Update Task Force (135), and White (136).

In summary, the reviewed studies provide evidence for the importance of ethics and bioethics training for professionals and students in the healthcare field. Despite existing limitations, specific bioethics training has proven effective in developing ethical knowledge, attitudes, values, and competencies. Both integrated curriculum-based training and external supplementary training have yielded positive results in enhancing ethical competencies. Intensive and ongoing programs have also shown favorable outcomes. However, continuous updating of these training is necessary to maintain their long-term impact.

## Conclusion

To conclude, the analysis of the 27 reviewed scientific articles in the field of medical ethics and bioethics reveals a lack of knowledge and skills to address ethical conflicts among healthcare professionals and students. Specific training in bioethics has been identified as an effective strategy to improve ethical knowledge, attitudes, values, and competencies in professional practice.

However, there is a lack of support from academic institutions and workplaces in implementing mandatory training programs in professional ethics. The importance of work experience and educational level as factors associated with improvement in ethical knowledge and values is highlighted.

Furthermore, significant differences were found in terms of ethical knowledge based on geographical region and healthcare discipline. Developed regions and certain disciplines showed better results in terms of ethical knowledge. These findings emphasize the need to consider regional and disciplinary specificities when designing ethical training programs.

A comprehensive approach is required to promote ethical training in the healthcare field. This involves incorporating medical ethics into academic curricula, providing continuous and effective training programs for practicing professionals, and addressing the specific needs of each regional and disciplinary context. Enhancing ethical and quality practice in the healthcare field is crucial to ensure the wellbeing of patients and the professional development of healthcare providers.

## Data availability statement

The original contributions presented in this study are included in the article/supplementary material, further inquiries can be directed to the corresponding author.

## Author contributions

All authors listed have made a substantial, direct, and intellectual contribution to the work and approved it for publication.
